# Prospective audit of the phenotype, causes and correlates of trachomatous and non- trachomatous trichiasis in a peri-elimination setting

**DOI:** 10.1371/journal.pntd.0011014

**Published:** 2022-12-27

**Authors:** Noopur Gupta, Praveen Vashist, Rachna Meel, Sumit Grover, Shubhi Jain, Deepak Kumar, Vivek Gupta, Radhika Tandon, Anthony W. Solomon

**Affiliations:** 1 Cornea, Cataract & Refractive Surgery Services, Dr. Rajendra Prasad Centre for Ophthalmic Sciences, All India Institute of Medical Sciences, New Delhi, India; 2 Community Ophthalmology, Dr. Rajendra Prasad Centre for Ophthalmic Sciences, All India Institute of Medical Sciences, New Delhi, India; 3 Oculoplasty Services, Dr. Rajendra Prasad Centre for Ophthalmic Sciences, All India Institute of Medical Sciences, New Delhi, India; 4 Department of Control of Neglected Tropical Diseases, World Health Organization, Geneva, Switzerland; London School of Hygiene&Tropical Medicine, UNITED KINGDOM

## Abstract

**Purpose:**

To explore the burden, clinical features and associations of trichiasis due to trachomatous and non-trachomatous aetiologies.

**Methods:**

Consenting patients presenting with trichiasis of either eyelid (of one or both eyes) attending the outpatient department, cornea and oculoplasty clinics of a tertiary eye care hospital in New Delhi between August 2018 to March 2020 were included. A comprehensive examination including visual acuity and anterior segment evaluation and photography was performed. Grade of trichiasis, laterality, presence and grade of entropion, and information on corneal opacity, conjunctival scarring, Herbert’s pits, and pannus, if present, were recorded in the case record form.

**Results:**

Overall, 302 patients (454 eyes) with trichiasis were recruited. The most common attributed cause of upper eyelid trichiasis (276 patients, 405 eyes) was trachoma (26% of patients), followed by Stevens-Johnson syndrome (23%), blepharokeratoconjunctivitis (17%) and old age (10%). A total of 296/405 eyes (73%) had some form of corneal involvement. Trachoma was not identified as the cause of trichiasis in any eye with lower eyelid-only disease.

**Conclusion:**

Only about a quarter of upper eyelid trichiasis in this peri-elimination setting was attributed to trachoma. A distinction between trachomatous and non-trachomatous trichiasis is imperative to meaningfully determine whether elimination of trachoma as a public health problem has occurred. These data may have implications for population-based estimates of TT prevalence in India and other peri-elimination settings.

## Author summary

“Elimination of trachoma as a public health problem” is defined in part by the presence of very low proportions of the population demonstrating the clinical sign “trachomatous trichiasis”. Trichiasis is the condition in which eyelashes touch the eyeball. But determining whether trichiasis is trachomatous in origin requires expertise that most graders who contribute to field-based surveys do not have; the right approach has therefore been debated. We undertook a twenty-month prospective study of trichiasis in a tertiary eye care hospital in New Delhi, using expert examiners. The setting is important here because India is very close to having each district demonstrate a prevalence of upper eyelid trichiasis lower than the agreed elimination threshold prevalence for trachomatous trichiasis. Our data suggest that only 26% of upper eyelid trichiasis in this setting was attributable to trachoma. Trachoma was not identified as the cause of trichiasis in any eye with lower eyelid-only disease, supporting the recent change in definition of trachomatous trichiasis to include only disease affecting the upper eyelid. However, better ways to routinely distinguish trachomatous from non-trachomatous trichiasis are still needed.

## Introduction

Trichiasis can be defined as inward turning of at least one eyelash so that it touches the eyeball, or evidence of recent epilation of in-turned eyelashes. It causes discomfort and can be classified as major and minor depending on the number of eyelashes touching the globe. If left untreated, it damages the ocular surface, in some cases causing corneal abrasion, ulceration and scarring, which leads to corneal opacity and hence impairment of vision [1].

Trachoma is one cause of trichiasis. Trachoma starts as a chronic keratoconjunctivitis caused by repeated episodes of infection with serovars A, B, Ba and C of *Chlamydia trachomatis* [2–3]. Recurrent episodes of conjunctivitis result in conjunctival scarring, which can eventually lead to misdirection of eyelashes and trichiasis [4–6]. Trachomatous trichiasis (TT) is the major risk factor for trachomatous corneal opacification, so treating trichiasis is central to preventing visual loss and blindness due to trachoma.

Though trachoma is an important cause, not all trichiasis should be attributed to trachoma. Trichiasis also occurs as a consequence of blepharokeratoconjunctivitis (BKC), Stevens-Johnson syndrome (SJS), chemical burns, ocular trauma, healed herpetic keratitis, other corneal infections, ocular surface disease and old age [7]. The existence of all these aetiological pathways for trichiasis is potentially very significant because “elimination of trachoma as a public health problem”, as defined by the World Health Organization (WHO), requires a reduction in the prevalence of TT unknown to the health system to <0.2% in adults aged ≥15 years, estimated through population-based surveys [8–9]. But survey examiners are not generally trained to determine the aetiology of trichiasis, and neither the absolute nor the relative burden of non-trachomatous trichiasis is known [10–11]. It is also unclear whether non-trachomatous trichiasis has the same sight-threatening potential as does TT.

To help national trachoma programmes understand the likely contribution of non-trachomatous trichiasis to the overall trichiasis burden in the community and investigate the potential association of non-trachomatous trichiasis with corneal opacification and vision loss, we studied patients with trichiasis in a peri-elimination setting.

## Methods

### Ethics statement

Ethical approval was obtained from the Institute Ethics Committee, All India Institute of Medical Sciences, New Delhi (IEC-251/04.05.2018, RP-31/2018) and the Ethics Review Committee, World Health Organization, Geneva, Switzerland (0003037). Participation in the study was at the discretion of each individual patient and written, informed consent was taken from each person (or their parent or guardian for those aged <18 years) before recruitment. Individuals who were not literate had the study documents read to them in a language that they understood, and their written consent obtained by witnessed fingerprint. Refusal to participate did not affect any patient’s standard of care. All responses were anonymized, with no personally identifiable information entered into the database except the hospital record number, which was used as a primary key, including for removing duplicate data entry.

A prospective audit was performed to collect data on patients presenting to the Dr. Rajendra Prasad Centre for Ophthalmic Sciences, a tertiary eye care hospital in New Delhi, India, with trichiasis of the upper or lower eyelid of one or both eyes. Between 1^st^ August 2018 and 18^th^ March 2020, all patients with trichiasis attending the Dr. Rajendra Prasad Centre’s outpatient department, cornea clinic or oculoplasty clinic were invited to participate. Their demographic profile including age, gender, and place of residence was recorded on the case record form (S1 Text). A structured history was taken. Clinical examination included estimation of uncorrected and best-corrected visual acuity (Snellen’s chart) and slit lamp biomicroscopy. Severity (minor: 1–5 trichiatic cilia touching the globe; major: six or more eyelashes touching the globe), location, and aetiology of trichiasis were noted for each eye. The phenotypic classification of trichiasis was based on characteristic clinical findings and relevant history (Table 1). Varied presentations of similar cases included under a specific etiology are defined in the table. For example, conjunctival scarring can be present in patients with both SJS and trachoma; however, the clinical presentation is very different for the two diseases. SJS is a dermatological emergency which is acute in onset with systemic features and usually occurs after oral intake of certain drugs. Trachoma, on the other hand, is disease in which scarring accumulates over years due to recurrent inflammation and fibrosis. Old age was inferred as a cause of trichiasis when no other cause was evident. It nearly always affected the lower eyelid; in some cases, both upper and lower eyelids were involved. For the purpose of the study, the aetiological diagnosis of BKC included cases with either blepharitis or keratitis or blepharokeratoconjunctivitis (Table 1). Photographs were captured for quality assurance and to facilitate later independent review of the clinical diagnosis by the investigators. Presence and grade of corneal opacity, entropion, and conjunctival scarring were recorded according to WHO’s FPC system; corneal pannus and Herbert’s pits were graded in accordance with other published schemes [12–14].

**Table 1:** Definitions used for etiologies of trichiasis/entropion.

Etiology of trichiasis	Definition used in this study
Trachoma^a^	**History**: poverty in childhood. **Examination**: previous follicular inflammation at the corneal limbus may have left depressions known as “Herbert’s pits”, which are traditionally said to be pathognomonic for previous active trachoma; Herbert’s pits are not invariably associated with trichiasis, however. Upper pole corneal pannus is also said to be a relatively specific marker for previous active trachoma. Visible conjunctival scarring in the upper tarsal conjunctiva and reduced tear volume are expected to accompany trichiasis. Tylosis and/or cicatricial entropion may be present. Focal trichiasis, restricted to a few eyelashes, may occur in minor cases without visible conjunctival scarring or entropion. In some cases with acute-on-chronic disease, papillary hypertrophy, other inflammatory responses or presence of extensive concretions may prevent visualization of underlying conjunctival scarring.
Stevens- Johnson syndrome	**History**: a dermatologic (and sometimes ophthalmological) emergency, usually occurring as a reaction to a newly-introduced medication. Characterized by a flu-like illness followed by the development of painful epidermal and mucosal bullous lesions with peeling, starting on the upper body and quickly spreading elsewhere. **Examination**: corneal or conjunctival epithelial defects, pseudomembranes, conjunctival hyperemia with purulent discharge, eyelid ulcers, corneal ulcer with thinning and perforation, anterior uveitis or panophthalmitis may be present. Late presentation is characterized by symblepharon, ankyloblepharon, subconjunctival scarring, eyelid margin keratinization, corneal scarring with vascularization and keratinization, trichiasis and entropion.
Blepharokeratoconjunctivitis	**History**: a chronic, inflammatory eyelid margin disease with secondary conjunctival and corneal involvement. **Examination**: the clinical spectrum of disease is varied, with findings ranging from inflamed eyelids, anterior lid margin telangiectasia, collarettes around the bases of the eyelashes, meibomian gland involvement, trichiasis and, in severe cases, corneal involvement that may include punctate erosions, punctate keratitis, phlyctenules, marginal keratitis, vascularization and ulceration.
Blepharitis^b^	**History**: inflammation of the eyelid margin causing ocular discomfort and irritation in a patient of any age and any ethnic group. The patient may describe painful swelling of the eyelid margins with redness, crusting and matting of eyelashes. Some patients have associated secondary dry eye disease. **Examination**: can be divided into anterior and posterior forms according to anatomic location, although there is considerable overlap, and both are often present. Anterior blepharitis affects the eyelid skin, base of the eyelashes and the eyelash follicles and includes the traditional classifications of staphylococcal and seborrheic blepharitis. Posterior blepharitis affects the meibomian glands and gland orifices and has a range of potential etiologies, the primary cause being meibomian gland dysfunction. Eyelid margin scarring may accompany trichiasis.
Keratitis	**History:** one or more previous episodes of active keratitis, experienced as eye pain and redness, watering, and photophobia, with or without history of trauma. **Examination:** a vascularized macular or leucomatous corneal opacity with or without iris adherence (adherent leucoma), and possibly focal trichiatic eyelashes due to previous inflammation. In cases of herpes zoster ophthalmicus, scars or hyperpigmentation of the skin (found unilaterally in the dermatome of the ophthalmic division of the trigeminal nerve) may also be evident.
Chemical injury	**History**: accidental or deliberate instillation of chemicals into the eyes. **Examination:** lid margin keratinization, symblepharon, eyelid deformity, corneal scarring and/or corneal vascularization are likely to accompany trichiasis. There may also be evidence of chemical injury to the skin.
Ocular trauma	**History**: trauma. **Examination**: corneal wound or scar, corneal vascularization, mydriasis, iridodialysis, traumatic iris defects, cataract or partially absorbed lens, phacodonesis, retinal tears or detachment, eyelid scars involving the eyelid margin with trichiatic eyelashes in the later stages. There may also be evidence of trauma elsewhere.
Senescence	**History**: old age. **Examination**: Presence of lower eyelid laxity and senile entropion accompanying the trichiatic eyelashes. Usually involves the lower eyelid.
Ocular surface disease^c^	**History**: generally, a chronic condition caused by poor quality or quantity of tears, which may cause the patient to complain of some combination of eye itching, redness, irritation, dryness and foreign body sensation. **Examination**: corneal xerosis, breakup of tears, decreased tear meniscus height, positive ocular surface staining.
Ocular cicatricial pemphigoid^d^	**History**: the patient may report previous congestion and inflammation of the eyelids. **Examination:** where trichiasis has supervened, accompanying findings include shortening of the fornices, symblepharon, ankyloblepharon, corneal scarring and keratinization, vascularization, eyelid margin thickening, loss of eyelashes, severe dry eye, and limbal stem cell deficiency.
Globe abnormality	**History**: injury or infection leading to catastrophic damage to the eyeball; traumatic or surgical enucleation or evisceration; congenital anophthalmos. Depending on the abnormality, there is likely to be no light perception in the affected eye or eyes. **Examination**: eyelid laxity, entropion and small, scarred or absent globe.
Congenital entropion/ epiblepharon	**History**: in-turned eyelashes since birth. In the case of epiblepharon, a family history consistent with autosomal dominant inheritance. **Examination**: in epiblepharon, a characteristic horizontal fold of skin and underlying pretarsal muscle overriding the margin of the upper or lower eyelid.
Eyelid tumour	**History**: an abnormal growth on the eyelid, which may or may not have been previously managed. **Examination**: mechanical ptosis, eyelid defects, eyelid shortening due to radiation or scarring, poliosis or madarosis. There may be other evidence elsewhere of the primary condition or its treatment.

^a^ An individual with trichiasis plus Herbert’s pits, upper pole pannus and/or conjunctival scarring does not necessarily have trachomatous trichiasis: Herbert’s pits, pannus and conjunctival scarring are associated with previous active trachoma and could be found as incidental findings in eyes with trichiasis of other causes.

^b^ Cases of trichiasis attributed to primary blepharitis were classified as being caused by BKC, not under ocular surface disease, which excluded patients with other primary ocular morbidity contributing to dry eye.

^c^ Cases attributed to ocular surface disease were those with primary dry eye disease, meibomian gland dysfunction, corneal xerosis and aqueous tear deficiency due to systemic autoimmune diseases such as Sjogren’s syndrome, systemic lupus erythematosus or rheumatoid arthritis; idiopathic filamentary keratitis; radiotherapy-induced dry eye; ocular surface alterations in patients with pterygium, limbal stem cell deficiency or chronic allergic conjunctivitis; blepharospasm and lid wiper keratopathy; ocular cicatricial pemphigoid, facial nerve palsy and neurotrophic keratopathy.

^d^ Cases attributed to ocular cicatricial pemphigoid were bracketed with those due to ocular surface disease, because ocular cicatricial pemphigoid is rare (two patients in the current series)

Departmental meetings were used to inform and engage all relevant clinical staff about the nature and conduct of the study. A two-day training programme equipped the study team with knowledge on the study’s methodology and operational aspects. Team members were trained by senior investigators about data collection procedures, the informed consent process, the completion of relevant paperwork, examination procedures including estimation of visual acuity, grading of trichiasis, conjunctival scarring, entropion, corneal opacity, upper pole pannus, and Herbert’s pits and the process for determining etiology of trichiasis. Ophthalmologists involved in the study all possessed intensive training in the cornea and anterior segment sub-specialty at the apex tertiary eye care institute of India and had extensive experience in identifying, diagnosing and managing trachoma and trichiasis in children and adults. Training used Objective Structured Clinical Evaluation (OSCE) techniques to quality-assure clinical evaluation. A teaching presentation with multiple photographs of each cause of trichiasis was used to discuss and deliberate on various causes and presentations of trichiasis, including how to differentiate the various phenotypes and record and grade the associated ocular findings of conjunctival scarring, entropion, corneal opacity, upper pole pannus and Herbert’s pits. Training also included a visit to the Cornea clinic and outpatient department in order to orient team members in clinical examination, photography and other procedures specific to the study. The first ten eligible consenting patients for each cause of trichiasis seen by any clinician were also seen independently by the investigators, and responses to the questionnaire by the investigators and treating clinician compared.

Training included an agreement analysis between the ophthalmologists, which was done as per standard recommendations wherein all study-related procedures were performed. Agreement of the graders with the OSCE slide set was conducted and inter-observer kappa values calculated. The minimum acceptable kappa between examiners was set at 0.8. Cases for assessing agreement were identified from the ophthalmic outpatient department of the Dr. Rajendra Prasad Centre for Ophthalmic Sciences.

### Statistical analysis

Data were transcribed from the original paper forms into a specially designed Epidata table with inbuilt validation and consistency checks. Analysis was carried out in Stata V.15.1 (Stata, College Station, Texas, USA). The primary outcome measures were distribution of cause of trichiasis and proportion of non-trachomatous trichiasis. In cases in which multiple aetiologies were observed, the eye- and person-level aetiological diagnoses were based on the disease considered more easily treatable or preventable.

Person-level descriptive analysis of specific etiologies, overall and stratified by age-groups, gender and location (upper, lower or both eyelids involved), was undertaken. Eye-level descriptive analysis of severity of trichiasis, entropion and conjunctival scarring in various specific etiologies was performed. Morphological features in eyes diagnosed with trachomatous and non-trachomatous trichiasis were compared using chi-square tests. A p value of <0.05 was considered statistically significant.

## Results

### Demographic profile of study patients

**Fig 1:**
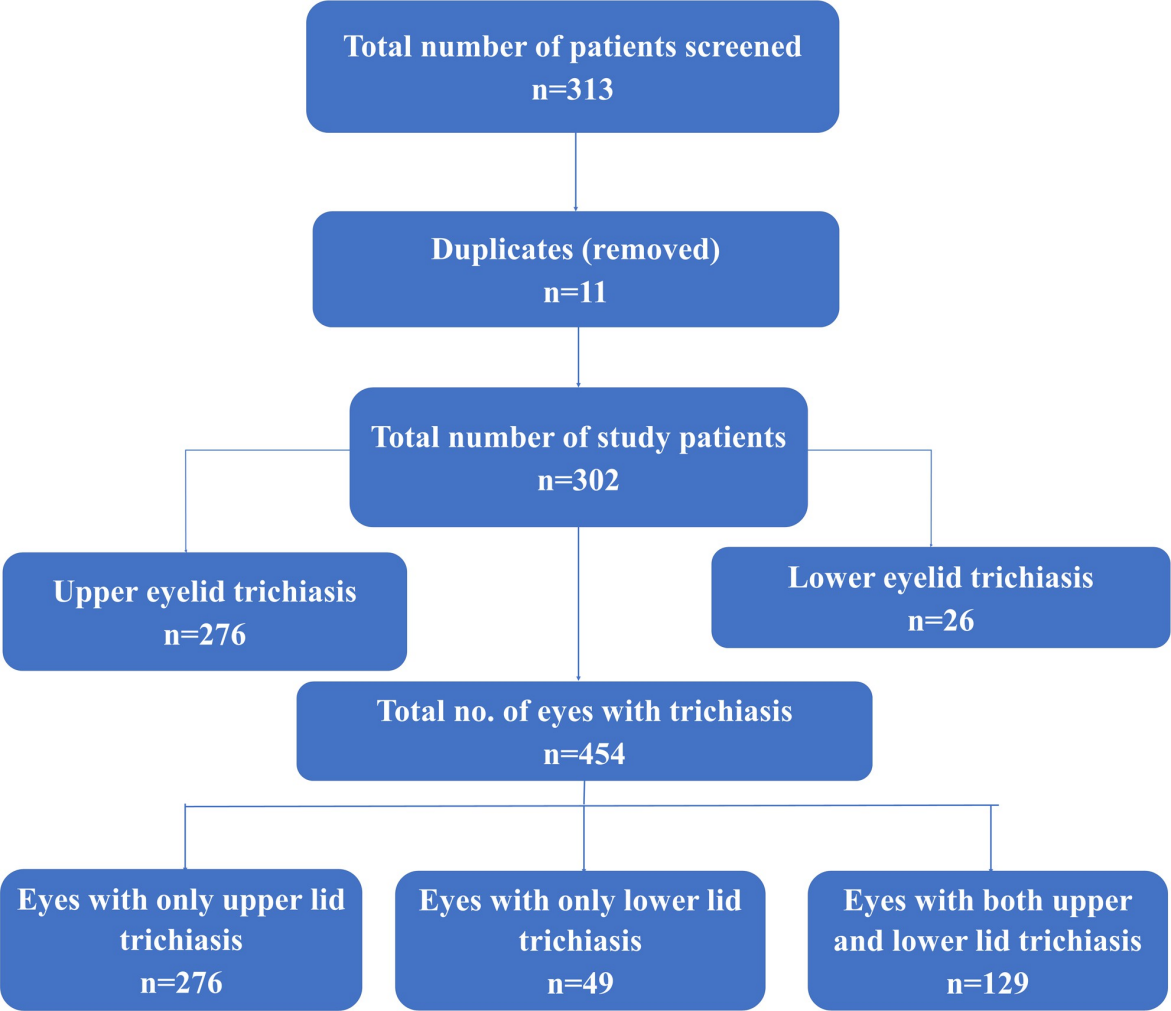
Flow chart depicting recruitment and characteristics of the study participants.

A total of 302 patients were recruited. Collectively, they had 454 eyes with trichiasis (Fig 1). The mean age of study participants was 50.0 (standard deviation 21.9) years. The age decade contributing the greatest number of patients was 60–69 years (28%) followed by 70–79 years (17%). There were more females (53%) than males (47%) in the series. This demographic profile is set out in more detail in Table 2.

**Table 2:** Aetiology of trichiasis by age and gender.

Age (yrs.)	Trachoma	SJS	BKC	Old age^c^	Ocular Surface Disease	Chemical Injury	Ocular Trauma	Globe Abnormality	Congenital	Lid Tumour	Unknown	Total, n (%)
**0–9**	0	0	2	0	0	2	1	1	6	0	0	12 (4)
**10–19**	0	10	5	0	6	3	1	1	0	0	1	27 (9)
**20–29**	0	16	6	0	1	5	4	1	0	0	0	33 (11)
**30–39**	0	17	6	0	1	3	0	0	0	0	0	27 (9)
**40–49**	4	5	6	0	2	2	3	1	0	0	1	24 (8)
**50–59**	6	5	6	4	2	2	2	2	0	1	0	30 (10)
**60–69**	30	8	15	18	5	0	4	1	0	0	3	84 (28)
**70–79**	28	2	6	14	1	0	0	0	0	0	1	52 (17)
**≥ 80**	5	1	3	1	1	0	1	0	0	0	1	13 (4)
**Gender**												
**Males**	30	30	32	9	7	12	9	4	4	1	3	141 (47)
**Females**	43	34	23	28	12	5	7	3	2	0	4	161 (53)
**Total, n (%)**	73 (24)	64 (21)	55 (18)	37 (12)	19 (6)	17 (6)	16 (5)	7 (2)	6 (2)	1 (0.3%)	7 (2%)	302 (100)

SJS = Stevens-Johnson syndrome; BKC = blepharokeratoconjunctivitis

^a^ The four patients in the 50–59 years age category were aged above 55 years

### Causes of trichiasis

**Fig 2:**
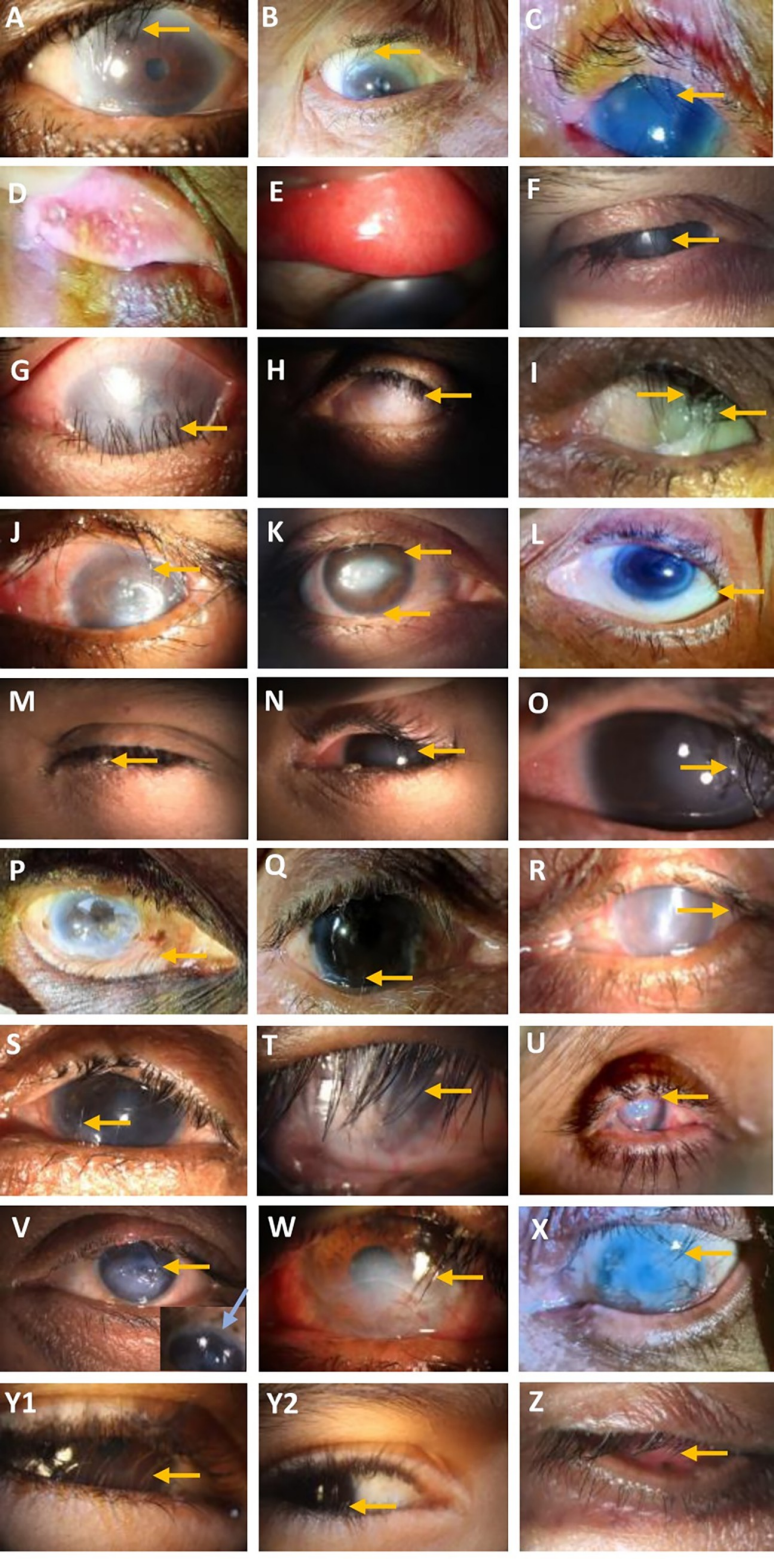
Photographs of selected patients showing different etiologies of trichiasis.

The most common cause of trichiasis was trachoma (24%), followed by SJS (21%), BKC (18%) and old age (12%). Clinical presentations and slit lamp photographs of patients with different etiologies of trichiasis are set out in Fig 2. Of 152 patients with bilateral trichiasis, 14 had different aetiologies in the two eyes (S1 Table). Table 2 presents a cross tabulation by age and cause of trichiasis. In those aged up to 39 years, BKC, SJS, ocular trauma and chemical injury were more common than trachoma. In ≥70-year-olds, trachoma was the dominant cause of trichiasis followed by old age.

### Upper eyelid trichiasis

**Fig 3:**
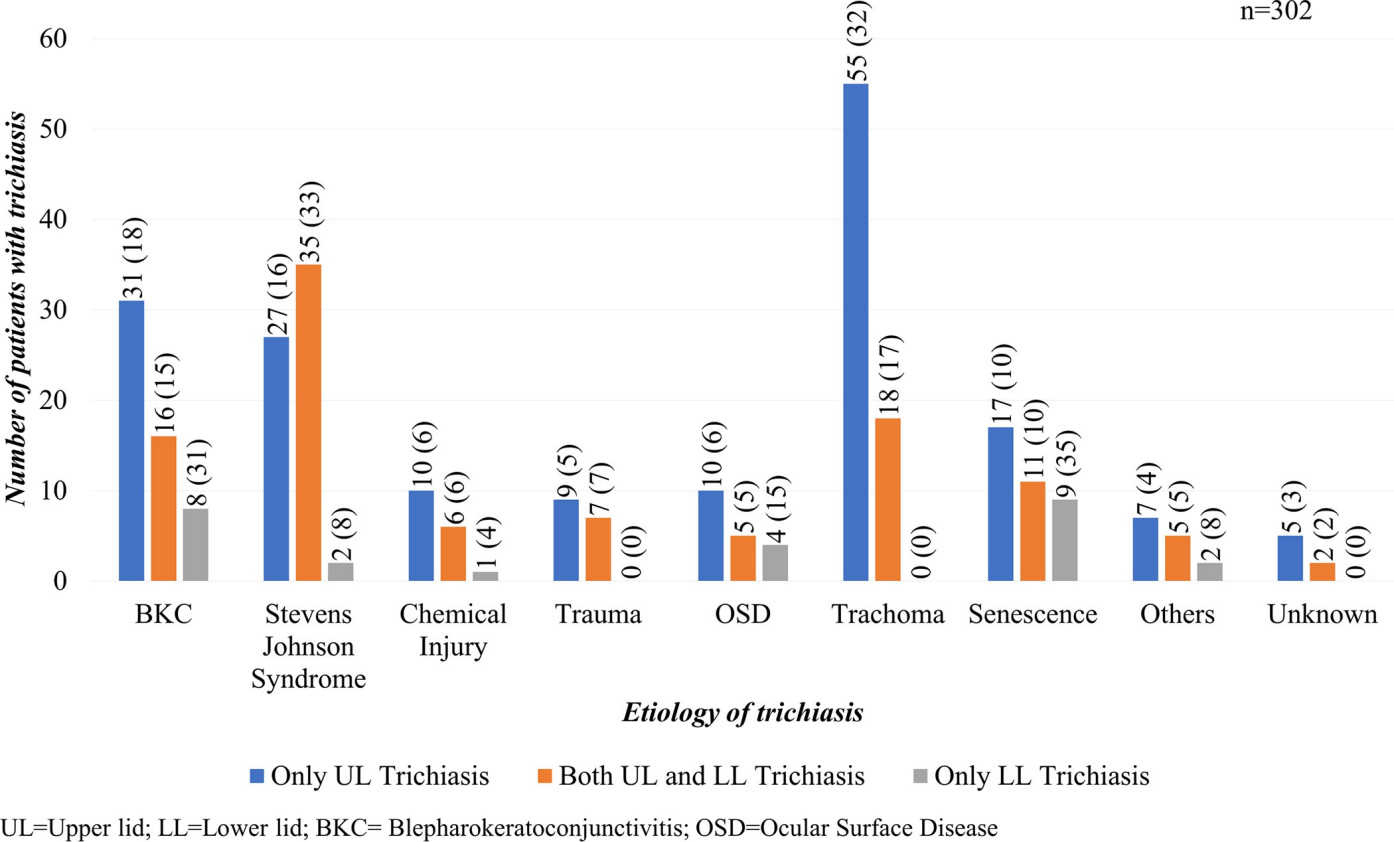
Aetiology of trichiasis in patients with upper and lower lid trichiasis.

**Fig 4:**
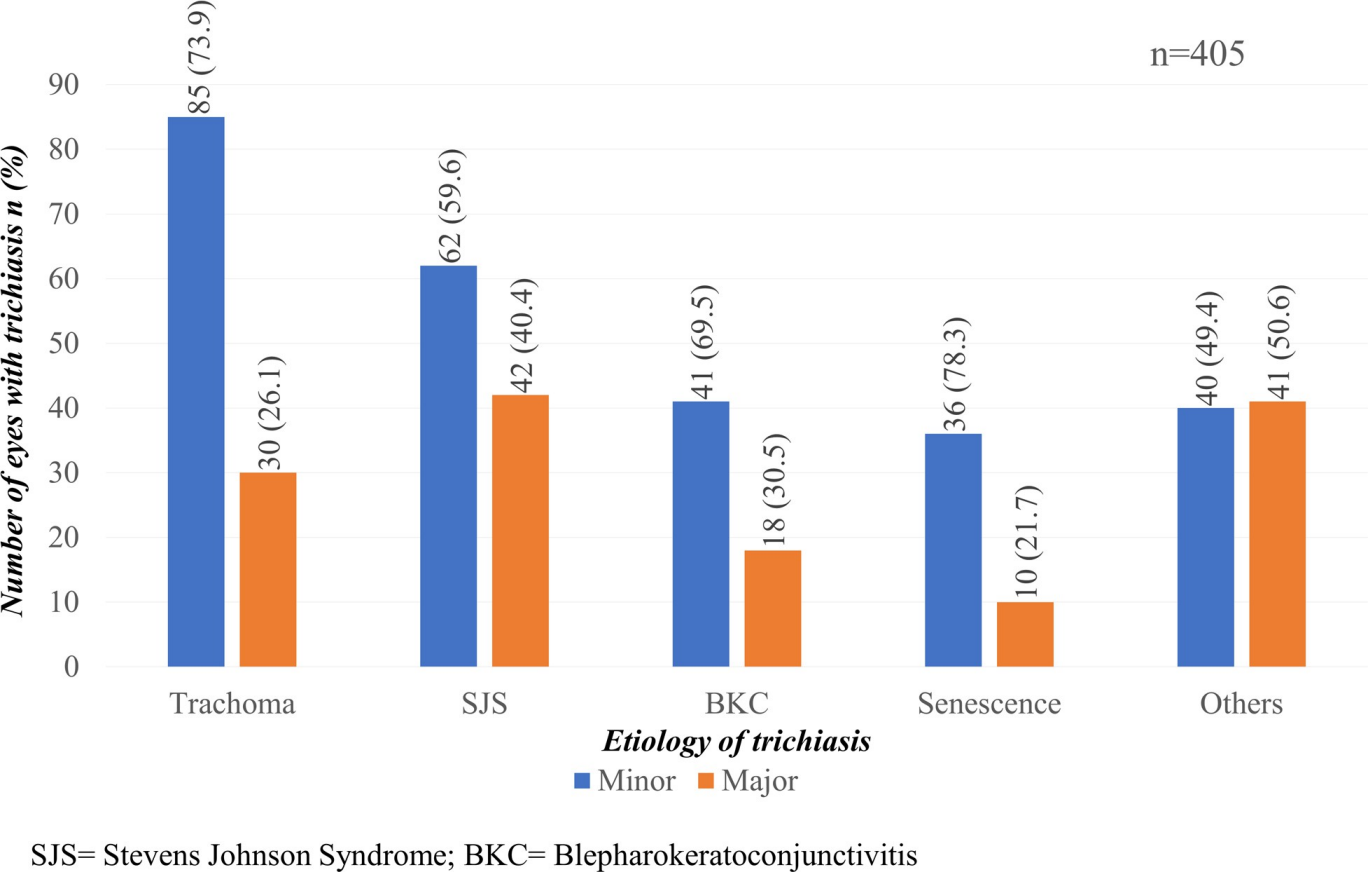
Severity of trichiasis in eyes with trachomatous and non-trachomatous trichiasis.

Among 276 patients who had upper eyelid trichiasis (with or without accompanying lower eyelid trichiasis), trachoma was the most common cause, being the aetiology identified in 73 patients (26%) (Fig 3). It was followed by SJS (62; 23%), BKC (47; 17%) and old age (28; 10%). Of 405 eyes with upper eyelid trichiasis, 115 (28%) had trachoma as the major cause followed by SJS (104 eyes, 26%). Most eyes with upper eyelid trichiasis had minor trichiasis (264/405; 65%) (Fig 4).

### Lower eyelid trichiasis

Of 26 patients with exclusively lower eyelid trichiasis, BKC (8; 31%), old age (9; 35%), and ocular surface disease (4; 15%) were the major contributory causes. Trachoma was not identified as the cause of trichiasis in any eye with lower eyelid-only disease.

### Duration of trichiasis

More than four-fifths of patients (83%) had had trichiasis for one to ten years prior to presentation. The mean duration of trichiasis due to trachoma (8 years) was greater than that of trichiasis due to SJS (7.1 years; P = 0.549), old age (5.6 years; P = 0.108) or BKC (4.2 years; P = 0.037).

### Morphological features

On detailed clinical examination, more than one-third of all eyes with upper eyelid trichiasis had mild conjunctival scarring (157/405; 39%), irrespective of the cause of trichiasis. Upper pole pannus was seen in eyes with both trachomatous (69/115; 60%) and non-trachomatous trichiasis (44/290; 15%); a majority of cases had only mild pannus Table 3.

**Table 3:** Morphological features in eyes with trachomatous and non-trachomatous trichiasis.

	Trachomatous Trichiasis n (%)^a^	Non-trachomatous Trichiasis n (%)^a^	Total n (%)^a^	P value^b^
**Upper pole pannus**				
0	45 (39)	238 (82)	283 (70)	
1	51 (44)	32 (11)	83 (20)	<0.001
2	15 (13)	5 (2)	20 (5)	
3	3 (3)	7 (2)	10 (3)	
Missing	1 (1)	8 (3)	9 (2)	
Total	115 (100)	290 (100)	405 (100)	
**Conjunctival scarring**				
0/indeterminate	24 (21)	92 (32)	116 (28)	
1	40 (35)	117 (41)	157 (39)	0.025
2	33 (28)	50 (17)	83 (20)	
3	17 (15)	30 (10)	47 (12)	
Missing	1 (1)	1 (0)	2 (1)	
Total	115 (100)	290 (100)	405 (100)	
**Herbert’s pits**				
0/indeterminate	46 (40)	268 (92.4)	314 (77)	<0.001
1	24 (21)	7 (2.4)	31 (8)	
2	20 (17)	7 (2.4)	27 (7)	
3	24 (21)	1 (0.4)	25 (6)	
Missing	1 (1)	7 (2.4)	8 (2)	
Total	115 (100)	290 (100)	405 (100)	
**Entropion**				
0	25 (22)	58 (20)	83 (21)	
1	44 (38)	59 (20)	103 (25)	<0.001
2	43 (37)	126 (44)	169 (42)	
3	3 (3)	47 (16)	50 (12)	
Total	115 (100)	290 (100)	405 (100)	
**Corneal opacity**				
Absent	29 (25)	80 (28)	109 (27)	0.628
Present	86 (75)	210 (72)	296 (73)	
Total	115 (100)	290 (100)	405 (100)	

^a^ Column percentage

^b^ Chi-square test

Trichiasis was associated with entropion in 322 eyes (80%): 103 (28%) had mild entropion, 169 (60%) moderate and 50 (12%) severe. As a group, eyes with non-trachomatous trichiasis had entropion of greater severity than eyes with TT (P<0.001). Evidence of previous eyelid surgery was seen in 46% (187 of 405) eyes with upper eyelid trichiasis. On subgroup analysis of non-trachomatous trichiasis eyes, eyes with SJS (P<0.001), BKC (P = 0.04), and chemical injury (P = 0.03) demonstrated higher grades of entropion than eyes with TT. There was no difference noted in severity of conjunctival scarring between eyes with TT and those with SJS (P = 0.157), and chemical injury (P = 0.181); however non-TT eyes with old age (P<0.001) and BKC (P = 0.004) as cause of trichiasis exhibited significantly milder or no conjunctival scarring when compared to eyes with TT.

### Corneal opacity in eyes with trichiasis

Corneal opacity (CO) was seen in 73% (296 of 405) eyes: mild CO in 19% (78 eyes), moderate in 15% (59 eyes) and severe in 39% (159 eyes). A total of 86 of 115 eyes (75%) with TT and 210 of 290 eyes (72.4%) with upper eyelid non-trachomatous trichiasis demonstrated evidence of corneal opacity. Rubbing of trichiatic eyelashes on the corneal surface had led, in the examiner’s opinion, to development of CO in 56% (45/115) of eyes with TT, while in eyes with upper eyelid non-trachomatous trichiasis, CO was related to presence of trichiasis in only 19% (40/210) of cases (P = 0.001).

### Visual acuity in patients with trichiasis

Across all 302 trichiasis patients, visual acuity was severely reduced (<3/60) in 104 patients (34%) while 121 had normal to mildly impaired visual acuity (40%). Presenting visual acuity was <3/60 in a majority of eyes with upper eyelid trichiasis (246/405; 61%). Similarly, best corrected visual acuity was <3/60 in the majority of eyes with upper eyelid trichiasis (239/405; 59%). The proportions of eyes with lower eyelid-only trichiasis that had uncorrected and best corrected visual acuities <3/60 were 49% (24/49) and 47% (23/49) respectively.

## Discussion

Though trachoma remains a major public health problem in some of the world’s poorest countries [15], in others, considerable progress has been made [16]. In India, population-based trachoma surveys conducted from 2014–2017 showed that the country had met the elimination target for active trachoma in all surveyed districts, but had a number of districts in which the prevalence of TT remained above threshold [17]; TT was defined using the WHO simplified system definition then in use, which included trichiasis in either the upper or lower eyelid[18]. Knowing what proportion of that apparent TT burden was actually due to trachoma could help to determine whether the disease really is a public health problem in any district of India. The present hospital-based study suggests that just less than a quarter of trichiasis (affecting either the upper or lower eyelid) in patients presenting to the Dr. Rajendra Prasad Centre is caused by trachoma.

Ophthalmologists undertook the examinations in this study. For routine population-based surveys to estimate TT prevalence, which are conducted door-to-door, health ministries are not generally able to employ examiners with similar levels of knowledge and experience to the examiners in our group. But such expertise is assumed not to be needed: aetiological diagnosis requiring clinical inference is not a part of routine surveys; instead, there has been an implicit assumption that in trachoma-endemic populations, all (or nearly all) trichiasis is due to trachoma. The present data show the extent to which that assumption is likely to be wrong as elimination as a public health problem is approached.

During the period in which this study was being conducted (and guided in part by an interim analysis of early data from it [7]), in order to address the problem of differentiating trachomatous from non-trachomatous trichiasis in the absence of expert examiners, WHO changed the definition of TT to focus only on the upper eyelid. The amended simplified grading system sign is, “at least one eyelash from the upper eyelid touches the eyeball or evidence of recent epilation of in-turned eyelashes from the upper eyelid” [19]. In our sample, 11% of patients had trichiasis only of one or both lower eyelids, without upper eyelid trichiasis in either eye. None of this lower eyelid-only disease was attributable to trachoma. This supports the WHO recommendation of including only upper lid trichiasis in the definition of TT. However, of the group of patients in our series with upper eyelid disease, still only 26% had TT. Better ways to differentiate TT from other trichiasis would be useful.

To this end, it has previously been proposed, including by co-authors of this manuscript [20], that absence of the WHO simplified system sign “trachomatous scarring” (TS, “presence of easily visible scarring in the upper tarsal conjunctiva [19]” could be taken to indicate that trichiasis is not of trachomatous origin. (TS equates with moderate [C2] or severe [C3] scarring in the more detailed grading system used in the present study.) We note that in our study population, 57% of trichiasis diagnosed as TT lacked concomitant moderate or severe conjunctival scarring despite scarring being specifically examined for, while 28% of non-trachomatous trichiasis had conjunctival scarring of this magnitude. The absence of conjunctival scarring in patients with minor trichiasis attributable to trachoma is a recognized phenomenon. Focal trichiasis, restricted to few cilia, may occur in mild cases without macroscopically visible conjunctival scarring of the tarsal conjunctiva and without entropion. In some patients with acute-on-chronic disease, papillary hypertrophy, other inflammatory responses or presence of extensive concretions may prevent the visualization of conjunctival scarring. We also understand that some conditions other than trachoma (SJS, chemical injury) cause trichiasis in the same way that trachoma does–by scarring the conjunctivae. What we did not expect was that TS would have such poor sensitivity (here 50/117; 43%) and specificity (210/290; 72%) for trachomatous disease amongst patients with trichiasis. It is also true that there is a problem with the subjectivity of the divisions between mild, moderate and severe scarring [12] (or, for TS, between “easily visible” and “not easily visible” scar [19]). Regardless, the fact remains that on the basis of these data, TS is unlikely to have value as a discriminator.

Eyes with non-trachomatous trichiasis demonstrated more severe entropion than eyes with TT. This could be due to the rapidly evolving nature of trichiasis in SJS and chemical injury, which together accounted for 35% of non-trachomatous cases. In contrast, in a peri-elimination setting in which the transmission intensity of ocular *C*. *trachomatis* has been low for many years, TT is unlikely to be commonly associated with severe entropion.

Herbert’s pits and upper pole pannus are often said, respectively, to be pathognomonic for and highly suggestive of trachoma [21,22]. In our sample, though definitely more common in eyes with TT than in eyes with non-trachomatous trichiasis, these signs were neither sensitive nor specific markers of aetiology at the individual level. Many eyes that have non-trachomatous trichiasis could, of course, also have Herbert’s pits and/or upper pole pannus due to previous trachoma, despite trachoma not being the primary cause of their trichiasis. (Application of these signs by appropriately-trained observers for the purpose of characterizing whole populations—rather than individuals—is a distinct exercise [23] and, we would argue, may still be appropriate.)

Our study design contains an inherent bias, in that it included only those individuals who found their way to specific clinics in a particular tertiary eye care hospital. Population-based trichiasis phenotyping studies from multiple trachoma endemic settings would yield higher quality data; however, this would be resource-intensive, requiring screening of huge sample sizes because of the low prevalence of trichiasis, particularly in peri-elimination populations such as ours. We contend that our data remain valuable because the hospital in question has an outstanding local, national and international reputation, and is therefore popular with patients and trusted by referring local healthcare providers. In other words, people with an eye problem tend to come through the doors if they can. Despite its location in the National Capital Territory of India, the hospital is immediately adjacent to a district (East Delhi) shown to have an above-elimination-threshold of trichiasis in the 2014–2017 survey series [17]. Our data are not necessarily generalizable to other contexts. This limits our conclusions, and we await similar investigations elsewhere in India and in other countries. Consideration could be given to having skilled examiners complete detailed phenotyping of patients with trichiasis identified in active case-finding exercises [24] or population-based prevalence surveys [25].

## Conclusion

Our data provide a detailed snapshot of cause-specific phenotypes of trichiasis in this part of India. We report that only 26% of upper eyelid trichiasis was attributable to trachoma in this setting. The data support the recent change in WHO’s definition of trachomatous trichiasis to include only disease affecting the upper eyelid. Further high-quality trichiasis phenotyping studies are needed from multiple trachoma-endemic populations around the world.

## Supporting information

S1 Text Case record form. (DOCX)

S1 Table Patients with trichiasis with different aetiologies in right and left eye. (DOCX)
