# Supplementary material for: Prospective audit of the phenotype, causes and correlates of trachomatous and non- trachomatous trichiasis in a peri-elimination setting
Source: PLoS Negl Trop Dis. 2022 Dec 27;16(12):e0011014. doi: 10.1371/journal.pntd.0011014 (PMC9829166; doi:10.1371/journal.pntd.0011014)
Supplement: S1 Text — (DOCX) [file pntd.0011014.s001.docx]

**Prospective audits of the phenotype, causes and correlates of trichiasis**

**Case Record Form**

| **A.** Patient UHID Number: | | | | **B.** STUDY NO: | |
| --- | --- | --- | --- | --- | --- |
| **C.** Age (years): |  |  | **D.** Gender: MALE FEMALE | | |
| **E.** District of current residence: | | | | |  |

| **FOR EACH EYE:** | | **RIGHT EYE** | **LEFT EYE** |
| --- | --- | --- | --- |
| **F.** Visual acuity: | |  |  |
| **F1.** UCVA | |  |  |
| **F2.** BCVA | |  |  |
| **G.** Is there upper eyelid trichiasis?  (Yes=1, No=2, 99=Others) | |  |  |
| **H.** Cause of upper eyelid trichiasis:  0= Unknown  1= Blepharitis  2= Stevens Johnson Syndrome  3= Chemical Injury  4= Trauma | 5= Ocular Cicatricial Pemphigoid  6= Trachoma  7= Senescence  8= Other (specify)  NA= No trichiasis |  |  |
| **I.** History of previous upper eyelid surgery for trichiasis on this eye?  (Yes=1, No=2, 99= Not Known) | |  |  |
| **Number of upper eyelid eyelashes epilated or touching the globe (with the eye in primary gaze)** | | | |
| **J.** Medial 1/3 of upper lid (nasal to nasal limbus) | |  |  |
| **K.** Middle 1/3 of upper lid (between nasal to temporal limbus) | |  |  |
| **L.** Lateral 1/3 of upper lid (between temporal to temporal limbus) | |  |  |
| **M.** Conjunctival scarring grade [FPC system]  0= No scarring on the conjunctiva/indeterminate  1= Mild: fine scattered scars on the upper tarsal conjunctiva  2= Moderate: more severe scarring but without shortening or distortion of the upper tarsus  3= Severe: scarring with distortion of the upper tarsus | |  |  |
| **N.** Entropion grade [Rajak 2011]  0= None  1= <50% of lid margin rolled inwards, without lash base–cornea contact  2= ≥50% of lid margin rolled inwards, without lash base–cornea contact  3= Lid margin rolled inwards with <50% lash base–cornea contact  4= Lid margin rolled inwards with ≥50% lash base–cornea contact | |  |  |
| **O**. Corneal opacity grade [FPC system]  0= Absent  1= Minimal scarring or opacity but not involving the visual axis  2= Moderate scarring or opacity involving the visual axis & pupillary margin  3= Severe central scarring or opacity with the pupillary margin not visible through the opacity | |  |  |
| **P.** If corneal opacity present, is it related to the trichiasis?  (Yes=1, No=2, 99= Not Applicable) | |  |  |
| **Q.**  If corneal opacity present but thought not to be related to the trichiasis, what is the probable cause of the corneal opacity? (free text) | |  |  |
| **R.** Height of upper pole pannus, measured from the upper limbus  0= <2.0 mm extension  1= 2.0 - <4.0 mm extension  2= 4.0 - <6.0 mm extension  3= 6.0 mm or more extension | |  |  |
| **S.** Herbert's pits  0= None/Indeterminate  1= One to three typical pits  2= More than three, but entire upper lunular not involved  3= Entire upper lunular involved  4= Cornea encircled or two rows of pits above the upper pole | |  |  |
| **T.** Is there lower eyelid trichiasis?  (Yes=1, No=2) | |  |  |
