# Supplementary material for: Prospective audit of the phenotype, causes and correlates of trachomatous and non- trachomatous trichiasis in a peri-elimination setting
Source: PLoS Negl Trop Dis. 2022 Dec 27;16(12):e0011014. doi: 10.1371/journal.pntd.0011014 (PMC9829166; doi:10.1371/journal.pntd.0011014)
Supplement: S1 Table — (DOCX) [file pntd.0011014.s002.docx]

**S1 Table** -Patients with trichiasis with different aetiologies in right and left eye

| **Serial No.** | **Age** | **Sex** | **Cause in Right eye** | **Cause in Left eye** | **Cause in Person** |
| --- | --- | --- | --- | --- | --- |
|  | 62 | M | Trachoma | BKC | Trachoma |
|  | 65 | F | Trachoma | Globe Abnormality | Trachoma |
|  | 75 | M | Trachoma | Phthisis bulbi | Trachoma |
|  | 21 | F | Stevens Johnson Syndrome | Unknown | Stevens Johnson Syndrome |
|  | 65 | F | Trauma | Stevens Johnson Syndrome | Stevens Johnson Syndrome |
|  | 62 | M | Old age | BKC | BKC |
|  | 82 | M | BKC | Globe Abnormality | BKC |
|  | 63 | F | BKC | Old age | BKC |
|  | 74 | M | BKC | Unknown | BKC |
|  | 56 | M | Old age | BKC | BKC |
|  | 61 | M | Old age | BKC | BKC |
|  | 73 | M | Globe Abnormality | BKC | BKC |
|  | 66 | M | Old age | Ocular Surface Disease | Ocular Surface Disease |
|  | 55 | M | Trauma | BKC | Ocular trauma |

M= Male; F=Female; BKC=Blepharokeratoconjunctivitis (includes cases with keratitis, blepharitis

and blepharokeratoconjunctivitis)
